# Generation and Characterization of Antibodies against Opioid Receptors from Zebrafish

**DOI:** 10.3390/ijms19010014

**Published:** 2018-01-02

**Authors:** Juan C. Arévalo, Enrique Hernández-Jiménez, Ada Jiménez-González, María Torres-Valle, Roman S. Iwasaki, Roger López-Bellido, Cristina Vicente-García, Raquel E. Rodríguez

**Affiliations:** 1 Department of Cell Biology and Pathology, Instituto de Neurociencias de Castilla y León (INCyL), Universidad de Salamanca, 37007 Salamanca, Spain; enheji@gmail.com (E.H.-J.); alumni.torresvallemaria@usal.es (M.T.-V.); roman.iwasaki@gmail.com (R.S.I.); crisvigar@usal.es (C.V.-G.); 2 Institute of Biomedical Research of Salamanca (IBSAL), University Hospital of Salamanca, 37007 Salamanca, Spain; adajg@usal.es (A.J.-G.); rogerlopez@usal.es (R.L.-B.); requelmi@usal.es (R.E.R.); 3 Department of Biochemistry and Molecular Biology, Instituto de Neurociencias de Castilla y León (INCyL), Universidad de Salamanca, 37007 Salamanca, Spain

**Keywords:** opioid receptors, antibodies, zebrafish

## Abstract

The opioid system is well conserved among species and plays a critical role in pain and addiction systems. The use of zebrafish as an experimental model to study development and genetics is extraordinary and has been proven to be relevant for the study of different diseases. The main drawback to its use for the analysis of different pathologies is the lack of protein tools. Antibodies that work in other models are not suitable for zebrafish due to the low degree of homology that exists among the opioid receptor protein sequences in different species. Here we report the successful generation and characterization of antibodies against the mu, delta 1 and delta 2 opioid receptors in zebrafish. The antibodies obtained, which are specific for each receptor due to the use of the C-terminus as antigens, work for Western blotting and immunohistochemistry. In addition, the antibodies against mu and delta 1 opioid receptors, but not those against delta 2, are able to immunoprecipitate the corresponding receptor from zebrafish lysates. The development of opioid receptor antibodies is an asset to the further study of the endogenous opioid system in zebrafish.

## 1. Introduction

The endogenous opioid system is an analgesic system implicated in the development of tolerance for and dependence on drugs such as opioids, cannabinoids, nicotine, and alcohol, and is thus of immense clinical relevance [1,2]. Opioids bind to the three classical opioid receptors, µ (MOR), δ (DOR) and κ (KOR). Of those, MOR plays a major role in mediating the effects of opioids—analgesia and addiction [3,4]—while DOR agonists produce weak analgesic effects [5,6]. Opioid addiction and misuse are responsible for many thousands of deaths in developed countries—more than 32,000 deaths in 2014 in the US [7]—resulting in a pandemic. Therefore, the adverse effects of opioids noticeably limit their optimal use in the clinical treatment of chronic pain conditions [8], and call for deeper and broader research to elucidate the mechanisms that control pain and addiction processes.

Zebrafish (*Danio rerio*) has been used successfully as a model organism to study disease-related pathways, given its easy in vivo manipulation [9]. Additionally, the visual accessibility of the inner organs during the larval stage make the zebrafish an excellent system for elucidating structural features of the opioid system, which could not be achieved in murine models. Previous studies have shown that in the endogenous opioid system of zebrafish, distinct opioid receptors are expressed: MOR, two delta opioid receptor duplicates (DOR1 and DOR2), one KOR and one opioid receptor-like (ORL) [10,11,12,13]. The expression pattern of these opioid receptors has been analyzed in the adult zebrafish brain using in situ hybridization [10,12,14]. In general, the opioid receptors show a wide pattern of distribution in the zebrafish nervous system, with the most intense labeling observed in regions like the spinal cord and in areas that are part of the analgesic and addiction pathways.

Great efforts have been made in the last few years trying to elucidate the effects of opioids and their receptors in relation to the crucial biomedical and social problems of pain and addiction. However, these studies are based on DNA or RNA methods, while events occurring at the protein level have been impossible to determine due to the lack of antibodies specific to zebrafish. Opioid antibodies raised against other species do not work with zebrafish, although other antibodies, like those for phosphorylated-Histone H3, function in the same way as in rodents [15,16]. Considering the importance of opioid research for the fields of pain and addiction, we have developed antibodies for the MOR, DOR1 and DOR2 from zebrafish.

## 2. Results

### 2.1. Alignment of Opioid Receptor Sequences

**Figure 1:**
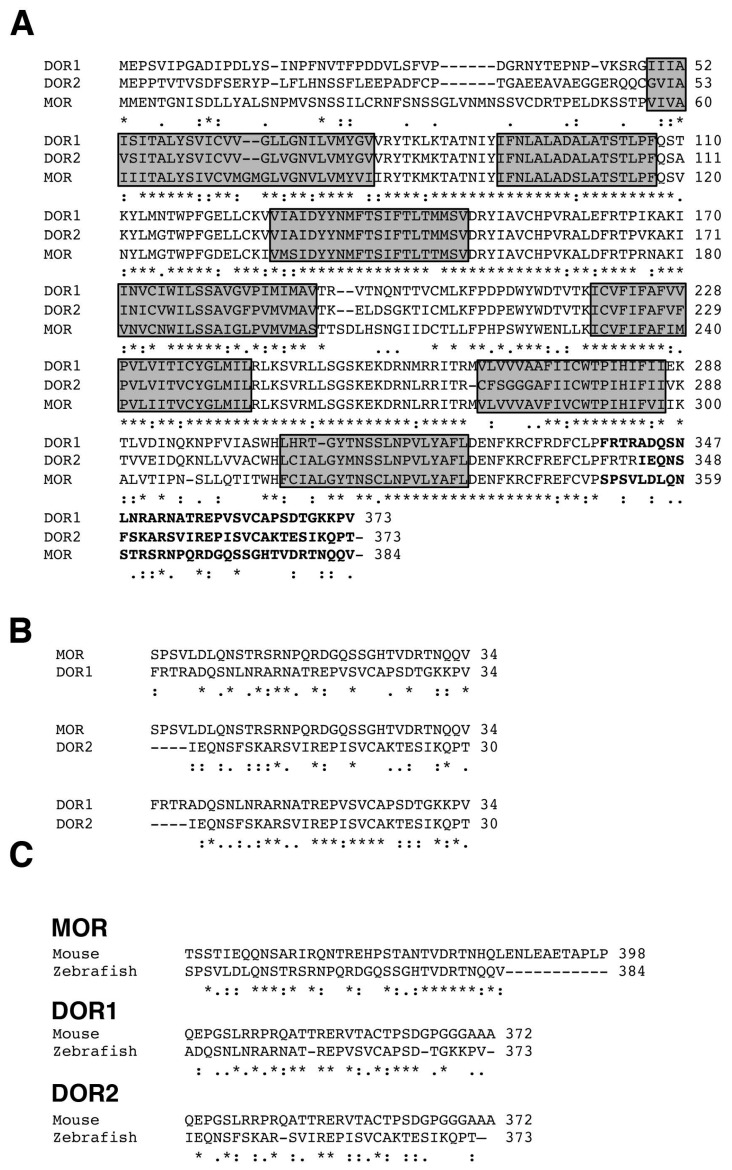
Alignment of opioid receptor protein sequences. (**A**) The protein sequences of µ (MOR) (NP_571782), δ1 (DOR1) (NP_571333) and δ2 (DOR2) (NP_997920) opioid receptors from zebrafish were aligned using the ClustalW2 program. Note that the N- and C-terminus are the least conserved regions among opioid receptors. In bold are the C-terminus sequences used to immunize rabbits. The asterisk (*) means that the residues are identical in all sequences in the alignment; the colon (:) means that conserved substitutions were observed; and the period (.) means that semi-conserved substitutions were observed. The shadow regions are transmembrane domains; (**B**) Alignment by pairs of the C-terminal sequences of opioid receptors from zebrafish. Note the low conservation in these sequences; (**C**) Alignment of the C-terminal sequences of different opioid receptors from mouse and zebrafish. Note the low conservation between the sequences of mouse and zebrafish.

To compare the sequences of the different zebrafish opioid receptors, we performed a ClustalW2 analysis (Available online: http://www.ebi.ac.uk/Tools/msa/clustalw2/) (Figure 1A). As can be observed, the N- and C-terminus are the most divergent regions among MOR, DOR1 and DOR2; whereas the regions corresponding to the transmembrane domains are well conserved among all opioid receptors (Figure 1A). Alignments between the C-terminus of MOR and DORs indicate very low conservation, similar to the conservation observed between the C-terminus of DOR1 and DOR2 (Figure 1B), suggesting that the antibodies raised against each antigen may discriminate between opioid receptor variants. Furthermore, alignments between MORs and DORs from mouse and zebrafish showed low conservation in the C-terminus (Figure 1C), suggesting that antibodies developed against mammal opioid receptors might not work in zebrafish. Considering that opioid receptors are glycosylated in the N-terminus [17,18], which may interfere with the production of the antibodies and/or detection of the native protein, we chose the C-terminus of the zebrafish MOR, DOR1 or DOR2 as immunogens to generate specific antibodies against each opioid receptor expressed in zebrafish.

### 2.2. Generation of Specific Zebrafish Opioid Receptor Antibodies

**Figure 2:**
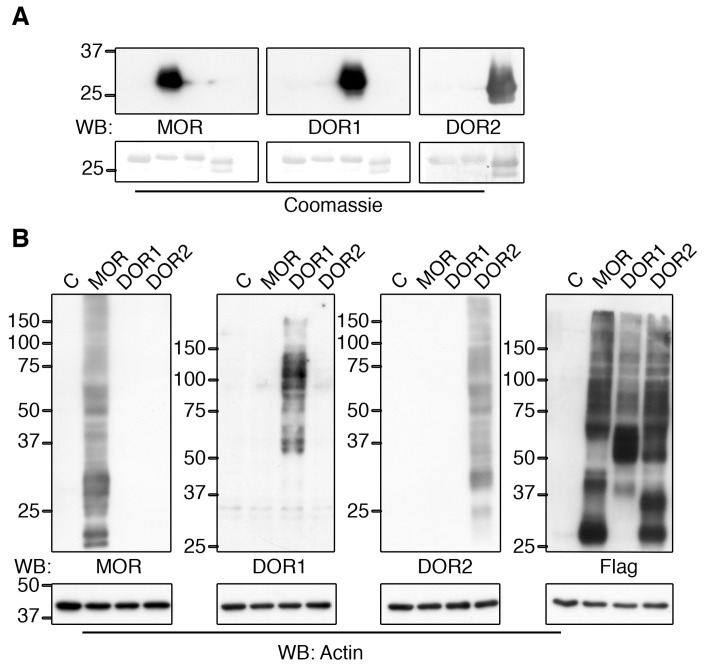
Reactivity and specificity of zebrafish opioid receptor antibodies. (**A**) Specific reactivity of opioid receptor antibodies against the antigen used to immunize rabbits. 3 µg of purified GST-WW, GST-MOR, GST-DOR1 and GST-DOR2 recombinant proteins were resolved using sodium dodecyl sulfate polyacrylamide gel electrophoresis (SDS-PAGE), transferred to membranes and incubated with purified antibodies that were pre-cleared of GST antibodies. Afterwards, membranes were stained with Coomassie to detect recombinant proteins. A representative experiment is shown (*n* = 4). Note the specificity of reactivity of each antibody against the corresponding antigen; (**B**) The antibodies recognized the corresponding zebrafish opioid receptor transiently expressed in HEK293 cells. HEK293 cells were transfected with the corresponding cDNA for MOR, DOR1 and DOR2 from zebrafish and Western blot analyses were performed with cell lysates. All the cDNAs transfected were Flag-tagged to identify their expression. Actin was used as a loading control. A representative experiment is shown (*n* = 3). Note the specificity in the reactivity of each antibody against the corresponding receptor.

Taking the above into consideration, we generated constructs fused to glutathione S-transferase (GST) with the sequences coding for the last 34, 35 and 30 amino acids of MOR, DOR1 and DOR2, respectively, in order to produce recombinant proteins in *Escherichia coli* bacteria. The generated GST-fusion proteins were purified and used as antigens to immunize rabbits as described in the Materials and Methods section. Purified antibodies were quantified and used to test their ability to recognize the corresponding antigen. To do so, 3 µg of each recombinant protein used to immunize the rabbits were loaded onto a sodium dodecyl sulfate polyacrylamide gel electrophoresis (SDS-PAGE) gel to perform Western blots. As a negative control, the GST fusion protein containing the WW domain of Nedd4-2 [19] was used. The incubation of each purified antibody with the Western blot membranes resulted in the recognition of the specific antigen (Figure 2A). None of the antibodies bound to the control indicating that the antibodies were reacting specifically against the protein fused to GST and not against GST (Figure 2A). To address whether the generated antibodies were able to bind to the full-length opioid receptor, each receptor from zebrafish was expressed in HEK293 cells and the lysates were subjected to Western blot analysis. Each antibody was able to specifically recognize the corresponding opioid receptor, but not the others (Figure 2B). The expression of the opioid receptors in HEK293 cells was monitored with Flag antibodies, since each protein was N-terminally tagged with the Flag epitope (Figure 2B, right panel). Therefore, we were able to generate antibodies using the C-terminal tail against MOR, DOR1 and DOR2 that show very strong specificity for each zebrafish opioid receptor in Western blot analysis.

### 2.3. The Generated Antibodies Recognize Zebrafish Opioid Receptors

**Figure 3:**
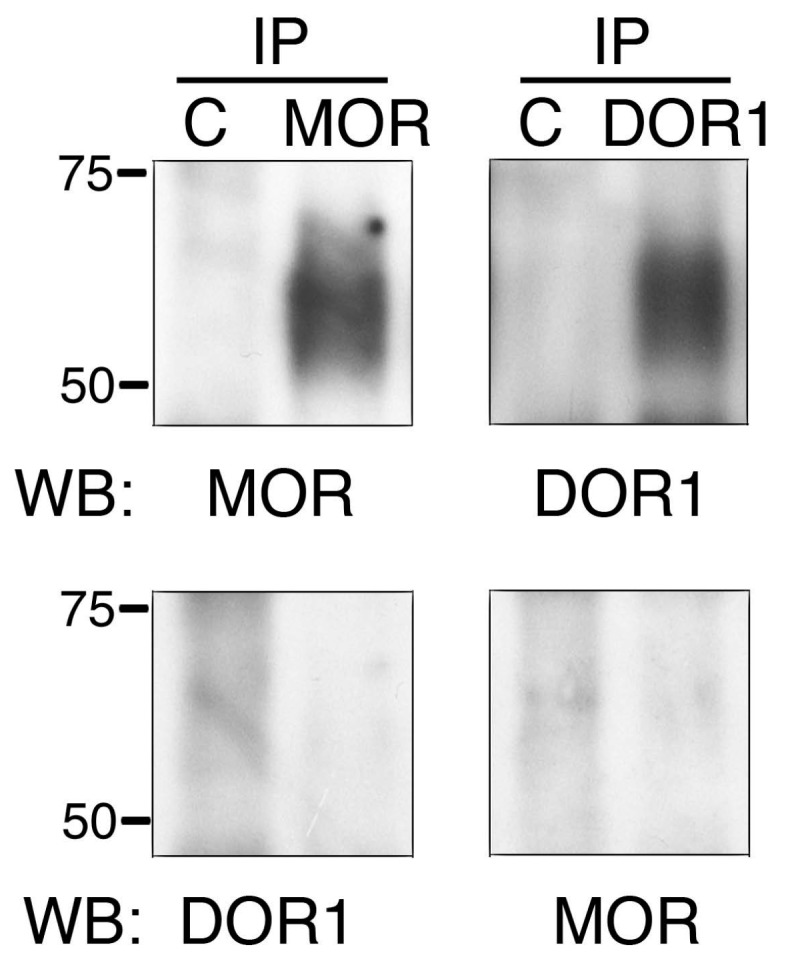
MOR and DOR1 antibodies immunoprecipitate the corresponding receptor from zebrafish embryos. Embryos 48 h post fertilization (hpf) were lysed and protein extracts were subjected to immunoprecipitation with 2 µg of MOR and DOR1 purified antibodies crosslinked to protein A agarose overnight at 4 °C. As a control, the same amount of purified rabbit IgGs was used. The immunoprecipitates were subjected to Western blot analysis. A representative experiment is shown (*n* = 3). Note that the signal was observed when the immunoprecipitates were incubated with the corresponding antibody (upper panels) but not with other antibodies (lower panels).

To assess whether the antibodies were able to bind the endogenously expressed opioid receptors, zebrafish embryos were lysed at 48 h post fertilization (hpf) and the lysates were subjected to immunoprecipitation. Considering that the size of opioid receptors is around 55–60 kDa, and that heavy chains from the antibodies may interfere with detection in the Western blot, we covalently bound the antibodies to protein A agarose beads prior to performing the immunoprecipitation. As a negative control, we coupled purified rabbit IgGs in the same way. After incubating the antibodies with zebrafish lysates, we performed Western blot analysis. As a result, we observed that MOR and DOR1 antibodies were able to immunoprecipitate the endogenous protein (Figure 3, upper panels). The use of control rabbit IgGs did not result in the immunoprecipitation of any opioid receptor (Figure 3, upper panels). No immunoprecipitated DOR2 was observed with the antibodies against this receptor. To further confirm the specificity of the MOR and DOR1 antibodies, we stripped the membranes corresponding to MOR or DOR1 immunoprecipitations and incubated them with DOR1 or MOR antibodies, respectively. We did not observe cross-reactivity of the antibodies against immunoprecipitated opioid receptors from zebrafish (Figure 3, lower panels). Therefore, we generated antibodies against MOR and DOR1 that were able to immunoprecipitate the endogenous proteins from zebrafish.

**Figure 4:**
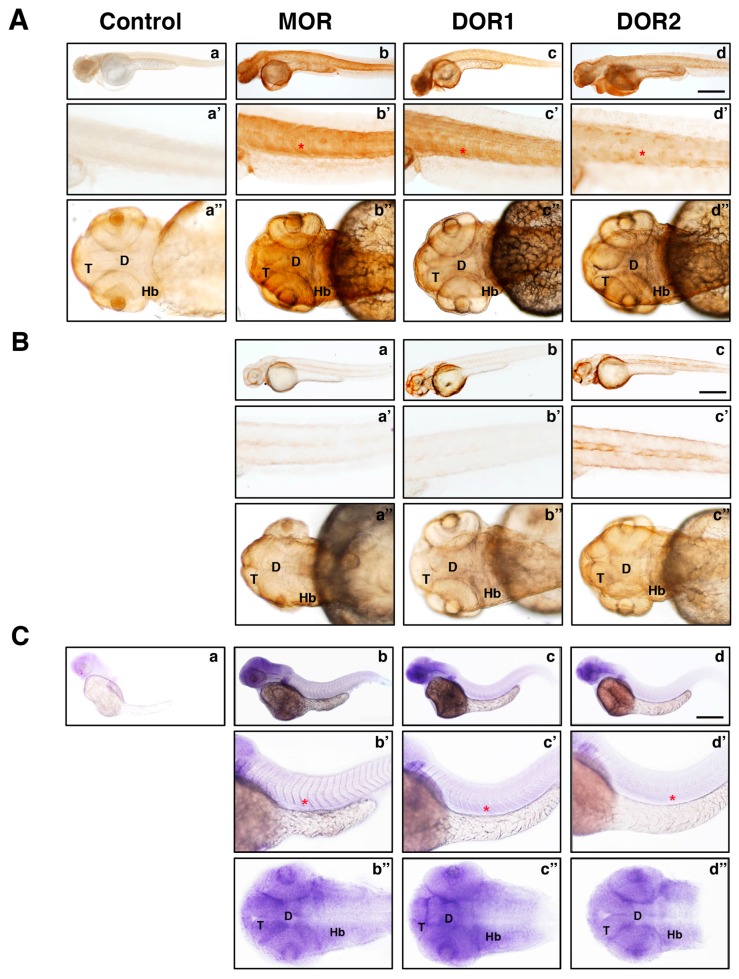
Antibodies against MOR, DOR1 and DOR2 recognize endogenous receptors in zebrafish embryos by immunohistochemistry. (**A**) Lateral and dorsal views of opioid receptors protein distribution in zebrafish embryos by whole-mount immunohistochemistry at 48 hpf. Embryos were processed as described in the Material and Methods section and subjected to immunohistochemistry with rabbit IgGs (Control), MOR, DOR1 or DOR2 antibodies for panels **a**, **b**, **c** and **d**, respectively. Magnified trunk (**a′**, **b′**, **c′** and **d′**) and head panels (**a**′′, **b**′′, **c′′** and **d′′**) are shown. Scale bar: 250 µm. Abbreviations: T: telencephalon; D: diencephalon; Hb: hindbrain; red asterisk: somite. A representative experiment is shown (*n* = 3); (**B**) Lateral and dorsal views of opioid receptors protein distribution in zebrafish embryos injected with the corresponding morpholino by whole-mount immunohistochemistry at 48 hpf. Embryos were injected with morpholinos specific to different opioid receptors at the one-to-four-cell stage in the yolk (panels **a**, **b** and **c**). Magnified trunk (**a′**, **b′** and **c′**) and head panels (**a**′′, **b**′′ and **c**′′) are shown. Scale bar: 250 µm. Note the reduced signal in embryos injected with the corresponding morpholino compared to non-injected embryos (panel (**A**)). A representative experiment is shown (*n* = 3). (**C**) Lateral and dorsal views of opioid receptors mRNA distribution in zebrafish embryos by whole-mount in situ hybridization at 48 hpf. Expression of opioid receptor mRNA using no probe (a) and antisense probes against MOR (b), DOR1 (c) and DOR2 (d) are shown. Magnified trunk (**b′**, **c′** and **d′**) and head panels (**b**′′, **c**′′ and **d**′′) are shown; red asterisk: somite. Scale bar: 250 µm. A representative experiment is shown (*n* = 3).

To evaluate whether the generated antibodies were suitable for immunohistochemistry in zebrafish, we used embryos at 48 hpf, which were then processed and incubated with the corresponding antibody as indicated in the Materials and Methods section. Embryos incubated with control antibodies did not show any staining (Figure 4A, panel a), whereas those incubated with different opioid receptor antibodies showed a staining (Figure 4A, panels b, c and d) that matched up with the corresponding mRNA expression (Figure 4C, panels b, c and d). To address the specificity of the antibodies for the corresponding protein, embryos were injected with morpholinos against MOR, DOR1 or DOR2 to knockdown the corresponding receptor. After incubation with the corresponding antibody, the signal previously detected (Figure 4A, panels b, c and d) was reduced when the morpholino was injected (Figure 4B, panels a, b and c), suggesting that the antibodies had specifically detected each opioid receptor. Together, these data indicated that the antibodies generated had indeed specifically detected each zebrafish opioid receptor.

**Figure 5:**
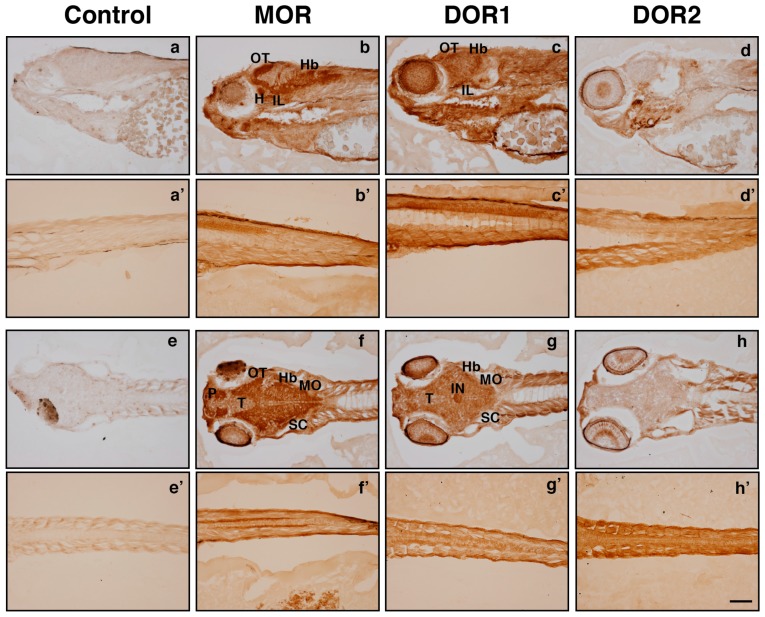
Antibodies against MOR, DOR1 and DOR2 recognize endogenous receptors in zebrafish larval sections by immunohistochemistry. Lateral (**a**, **b**, **c**, **d**) and dorsal (**e**, **f**, **g**, **h**) views of opioid receptors protein distribution in head and spinal cord sections from zebrafish larvae 5 days post fertilization. Sections were obtained as described in the Materials and Methods section and subjected to immunohistochemistry with rabbit IgGs (Control), MOR, DOR1 or DOR2 antibodies. Abbreviations: hypothalamus (H), hindbrain (Hb), inferior lobullum (IL), interpeduncular nucleus (IN), medulla oblongata (MO), optic tectum (OT), pallium (P), semicircular canal (SC) and telencephalon (T). A representative experiment is shown (*n* = 3). Scale bar: 100 µm. A representative experiment is shown (*n* = 3).

To identify the specific regions in the CNS where opioid receptors are expressed, we performed immunohistochemistry experiments in sections from 5 days post fertilization (dpf) zebrafish larvae. We observed that our developed antibodies detected high MOR expression in the optic tectum (OT), cerebellum (Cb), medulla oblongata (MO), telecenphalon (T), pallium (P), hypothalamus (H) and inferior lobullum (IL) (Figure 5). DOR1 was also expressed in the OT, Cb, H and IL, and highly expressed in the interpeduncular nucleus (IN), whereas the reactivity observed for DOR2 was more diffuse in the whole brain (Figure 5). In addition, a strong labeling was observed in the spinal cord with all three antibodies, similar to previously reported [10,12,14]. Together, this data indicated that the generated antibodies recognized the specific expression of the different opioid receptors in zebrafish.

## 3. Discussion

Despite a plethora of studies investigating the mammalian opioid system, many issues regarding opioid regulation remain unknown. Zebrafish have been used to study the neurobiology of drug addiction, withdrawal and reward in ways that cannot be fully established in other animal models [20,21,22,23,24,25]. In this way, zebrafish show advantages compared to other vertebrate animal models in various aspects: maintenance of the populations is inexpensive, the embryos develop rapidly, are transparent, permeable to drugs, and easily genetically manipulated [9,26]. Furthermore, zebrafish embryos develop externally, unlike mammalian embryos that develop in the uterus, and therefore are not influenced by the mother’s biochemical processes or exposure to drugs. Thus, the effects of opioids on embryos can be observed more directly, which will help to untangle the complex interactions that leads to the fetal defects observed after maternal opioid consumption. [27,28]. Besides, Malafoglia et al., (2014), recently used zebrafish larvae to show at the molecular level that they respond to stimuli that induce inflammation and axonal degeneration similar to mammals, showing that zebrafish is a good model for the study of the endogenous mechanisms that describe pain [29].

One difficulty in the use of zebrafish as a model for studying different functions, including the opioid system, is the lack of proper tools, such as antibodies. Most antibodies have been developed against human, rat or mouse proteins, and do not recognize their zebrafish homologues. Indeed, Stevens previously reported that mammal and zebrafish opioid receptors were very divergent [30]. Therefore, it is highly important to develop antibodies against opioid receptors that work in zebrafish. Here, we describe the production and the characterization of antibodies against the opioid receptors from zebrafish. We have utilized standard methodology to identify regions in the opioid receptors that were divergent among them looking for selectivity. The antibodies were raised against the C-terminal tails of the receptors as these regions have a low similarity among MOR, DOR1 and DOR2. These antibodies recognize the antigen as well as the corresponding full-length opioid receptor expressed in HEK293 cells in a very specific manner. Furthermore, the antibodies against MOR and DOR1, but not DOR2, are able to immunoprecipitate the corresponding opioid receptor from zebrafish embryo lysates. All antibodies were able to stain whole embryos and larval sections in a pattern that resembles the one observed in the adult zebrafish brain using in situ hybridization [10,12,14]. Hence, using classical, standard biochemical methods to generate and purify antibodies, new antibodies have been successfully developed that will allow the study of opioid receptors in zebrafish. The bottleneck represented by the lack of antibodies recognizing many different zebrafish proteins could be overcome with the approaches utilized as demonstrated in this article.

## 4. Materials and Methods

### 4.1. Plasmids

The 3′ DNA fragments corresponding to the nucleotides 1051–1152 of MOR (NM_131707), 1015–1122 of DOR1 (NM_131258) and 1030–1122 of DOR2 (NM_212755) from zebrafish were amplified from cDNA by polymerase chain reaction (PCR) using primers flanked by *EcoRI* and *XhoI* sequences and cloned in the pGEX-6P-1 vector. These plasmids were expressed in bacteria in the presence of IPTG (0.1 mM) to generate recombinant proteins consisting of GST fused to the last 34 (S351-V384), 35 (F339-D373) and 30 (I344-T373) amino acids of MOR, DOR1 and DOR2, respectively. The cDNAs of *MOR*, *DOR*1 and *DOR*2 were amplified from zebrafish cDNA by PCR using primers flanked by *NotI* and *XhoI* restriction site sequences and cloned in the pFlag-CMV-1 vector.

All DNA constructs were verified by sequencing.

### 4.2. DNA Transfections

Plasmids containing cDNAs of *MOR, DOR*1 and *DOR*2 were transiently transfected into HEK293 cells using the calcium-phosphate method [31].

### 4.3. Western Blot Analysis and Immunoprecipitation

Western blot analyses were performed as previously described [32]. Cells were lysed in a lysis buffer (10 mM Tris, pH 7.4, 150 mM NaCl, 2 mM ethylenediaminetetraacetic acid, 1% NP-40, 1 mM phenylmethylsulfonyl fluoride, 1 μg/mL Aprotinin, 2 μg/mL Leupeptine, 1 mM Vanadate, 10 mM NaF and 20 mM β-glycerophosphate) and zebrafish embryos were lysed in the same buffer, supplemented with 0.1% sodium docecyl sulfate, for 45 min at 4 °C with gentle shaking, and centrifuged at 14,000× *g* for 15 min to eliminate cell debris. For immunoprecipitations, 300 µg of zebrafish embryo lysates were incubated with 2 µg of the corresponding antibody covalently coupled to protein A-agarose at 4 °C for at least 4 h with gentle shaking. SDS-buffer was added to the lysates or immunoprecipitates and they were boiled for 7 min to denature the proteins. Proteins were resolved by SDS-PAGE and Western blots were performed with antibodies against the different proteins. To prevent interferences with the immunoglobulin chains, we used ProtA-conjugated with horseradish peroxidase when the same species of antibodies were used for both immunoprecipitations and Western blots.

### 4.4. Recombinant Protein Production

The production of GST-fusion recombinant proteins was done in DH5α *E. coli* bacteria. Briefly, bacteria transformed with the corresponding plasmid were inoculated in Luria broth media supplemented with ampicillin (100 µg/mL) overnight at 37 °C. The culture was then diluted 1/100 with fresh media and allowed to grow at 37 °C to the exponential phase (Optic Density_600_ ≈ 0.4–0.6), when the expression of recombinant protein was induced with IPTG (0.1 mM) for 3 h. Bacteria were harvested, resuspended in ice-cold phosphate-buffered saline supplemented with lysozyme and protease inhibitors and sonicated to break the cells. Triton X-100 was added to the sonicated solution and centrifugation was performed to remove the debris. The supernatant was incubated with glutathione beads overnight at 4 °C. The following morning, beads with the bound recombinant GST-tagged protein were gently collected and washed extensively with ice-cold PBS. GST fusion proteins were eluted from the beads with a solution containing 0.1 M glycine pH 2.5, which was later on neutralized with 1 M Tris pH 9.5 and supplemented with NaCl to a final concentration of 20 mM. To generate the affinity columns to purify antibodies, recombinant proteins bound to glutathione beads were covalently crosslinked with dithiobis(succinimidyl propionate) reagent (Sigma-Aldrich, St. Louis, MO, USA) following the instructions of the manufacturer.

### 4.5. Immunization of Rabbits

Female rabbits (New Zealand) were initially immunized with 0.5 mg of the recombinant protein mixed with complete Freund adjuvant. Boost immunizations were performed with 0.25 mg of recombinant protein mixed with incomplete Freund adjuvant at 14, 28, 49, 70 and 91 days after the first immunization. Sera were collected at 0 (pre-immune), 38, 59, 80 and 101 days after the first immunization. All procedures used in this work were in accordance with the guidelines of the European Communities Council Directive 2010/63/UE and the RD 53/2013 Spanish legislation for the use and care of animals. The experimental protocol (#63; 26 November 2007) was approved by the Bioethics Committee of the University of Salamanca.

### 4.6. Purification of Antibodies

GST protein crosslinked to glutathione beads was used as an affinity column to pre-clear sera of GST antibodies. GST-MOR, GST-DOR1 and GST-DOR2 recombinant proteins crosslinked to glutathione beads were used as affinity columns to purify specifically the corresponding antibodies from sera previously incubated with the GST-column. Antibodies bound to the affinity column were eluted using a solution with 0.1 M glycine pH 2.5. The solution of eluted antibodies was neutralized with 1 M Tris pH 9.5 and supplemented with NaCl to a final concentration of 20 mM. Purified antibodies were quantified and tested in Western blots.

### 4.7. Immunohistochemistry on Zebrafish Embryos

All experiments regarding zebrafish were performed using the wild-type AB strain. Zebrafish embryos 48 hpf were fixed in 4% paraformaldehyde. After extensive washing with PBS, the embryos were blocked in PBS + 5% Normal Goat Serum + 0.05% Triton X-100 for 1 h and incubated with the corresponding antibody (2.5 µg/mL) for 2 days at 4 °C with gentle rocking. The embryos were washed three times with PBS, incubated with biotinylated goat anti-rabbit antibody (1/500) for 2 h, washed three times with PBS, and incubated with ABC solution (Vectastatin Elite ABC Peroxidase, Vector Cat# PK-610) for 30 min. Then, three washes with PBS were carried out and the staining was developed with ImmPACT DAB substrate (Vector Cat# 4105). Images were taken with an Olympus AX70 microscope equipped with an Olympus DP70 camera.

### 4.8. Immunohistochemistry on Zebrafish Sections

Zebrafish larvae 5 dpf were fixed in 4% PFA for 24 h at 4 °C, washed with PBS embedded in agarose and the blocks were incubated with 30% sucrose until they sank. Blocks were frozen at −20 °C until they were sectioned on a cryostat at 20 µm. Sections were allowed to dry O/N at room temperature (RT), washed with PBST (PBS, 0.1% Triton X-100), PBST with 25%, 50% and 75% Methanol and then with 100% Methanol at −20 °C for 1h. Sections were washed with PBST with 75%, 50% and 25% Methanol. After washing twice with PBST, the sections were blocked in PBST + 10% DMSO + 10% NGS for 2 h at RT and incubated in with PBST + 5% normal goat serum the corresponding antibody (4 µg/mL) for at least 2.5 days at 4 °C. The sections were washed twice with PBS for 10–15 min, incubated with biotinylated goat anti-rabbit antibody (1/500) in PBST + 5% NGS for 2 h, washed twice with PBS for 10–15 min and incubated with ABC solution (Vector) for 30 min. Then, two washes with PBS for 10–15 min were carried out, and the staining was developed with ImmPACT DAB substrate (Vector). Images were taken with an Olympus AX70 microscope equipped with an Olympus DP70 camera.

### 4.9. Morpholino Microinjection

Morpholino antisense (MOs) oligomers were used for opioid receptor knockdown (Gene Tools, LLC., Philomath, OR, USA). The sequences used to knockdown the opioid receptors were MOR (AATGTTGCCAGTGTTTTCCATCATG), DOR1 (GAATGACGGACGGCTCCATCGCTTC), and DOR2 (GGAGGCTCCATTATGCTCGTCCCCT). These sequences have been previously reported and validated [33,34]. These MOs were diluted in sterilized water to a stock concentration of 0.3 mM and the concentrations employed for the different opioid receptors were 0.2 µM (MOR), 1 µM (DOR1) and 1 µM (DOR2). The MO experimental groups were MOR, DOR1 and DOR2. Approximately, 3 nL of each MO was microinjected into the yolks of zebrafish embryos at the one-to-four-cell stage according to published protocols [35] with a micromanipulator-microinjector system from Eppendorf AG (Hamburg, Germany).

### 4.10. mRNA Probes and Whole-Mount in Situ Hybridization

Riboprobes. pcDNA3 Plasmids containing cDNAs for *MOR, DOR1* and *DOR2* (nucleotides 1–1155 of MOR ref NM_131707, 1–1122 of DOR1 ref NM_131258, and 1–1122 of DOR2 ref NM_212755) were used to synthesize digoxigenin-labelled antisense (using SP6 RNA polymerase) and sense (using T7 RNA polymerase) riboprobes as previously described [36]. Riboprobes were used at a final concentration of 0.5 µg/mL.

Embryos at 48 hpf were dechorionated, fixed with 4% PFA in PBS overnight at 4 °C, washed twice in PBS for 5 min each at room temperature (RT). Then embryos were rehydrated in consecutive dilutions of methanol/PBS (75%, 50% and 25%), 5 min for each dilution. Then they were washed 4 times for 5 min each in 100% PBS-Tween 20 (0.2%) (PBT). Proteinase K (10 µg/mL) was used to permeabilize the embryos at RT over 40 min. Proteinase K digestion was stopped by incubating the embryos for 20 min in 4% (*wt*/*vol*) (PFA) in PBS. Four washes, 5 min per wash, in PBT were performed to remove residual PFA. After 2 h of prehybridization the embryos were left overnight at 64 °C to hybridize (with *MOR*, *DOR1*, and *DOR2* antisense and sense riboprobes, each one separately). Washes were performed every 20 min in each wash solution with prehybridization/Tris-buffered saline (TBS) (50%/50%) and TBS-Tween 20 (0.2%) (TBST) over 2 h. Then, the embryos were blocked with blocking buffer (goat serum + TBST) for 2 h and were incubated overnight with antidigoxigenin antibody conjugated with alkaline phosphatase (1:3000, Roche) at 4 °C. The following day, the embryos were washed with Xpho solution (1 M Tris HCl, pH 9.5, 1 M MgCl_2_, 4 M NaCl and 20% Tween-20) for 10 min over 1 h, and finally the hybridization was developed with a fresh NBT/BCIP mix (Roche, Basel, Switzerland).
